# A Step Forward in the Characterization of Primary Brown Trout Hepatocytic Spheroids as Experimental Models

**DOI:** 10.3390/ani13142277

**Published:** 2023-07-12

**Authors:** Rodrigo F. Alves, Célia Lopes, Eduardo Rocha, Tânia V. Madureira

**Affiliations:** 1 Team of Animal Morphology and Toxicology, Interdisciplinary Centre of Marine and Environmental Research (CIIMAR/CIMAR), University of Porto, Terminal de Cruzeiros do Porto de Leixões, Av. General Norton de Matos s/n, 4450-208 Matosinhos, Portugal; rodrigo.r.f.alves@hotmail.com (R.F.A.); cclopes@icbas.up.pt (C.L.); erocha@icbas.up.pt (E.R.); 2 Laboratory of Histology and Embryology, Department of Microscopy, ICBAS—School of Medicine and Biomedical Sciences, University of Porto, Rua Jorge Viterbo Ferreira 228, 4050-313 Porto, Portugal

**Keywords:** brown trout, primary hepatocytes, spheroids, 3D cultures

## Abstract

**Simple Summary:**

The liver is a vital organ for xenobiotic biotransformation and a prime target for drug toxicity. The search for alternative experimental liver models in fish and other species is critical to reducing the number of individuals used in animal experiments. Mammal liver spheroids have emerged as a viable in vitro model with similar morphofunctional properties to the liver. These three-dimensional (3D) structures are not well studied in fish. In this study, primary brown trout hepatocyte spheroids were characterised in biometry, histomorphology, and basal expression of a selection of target genes (metabolism and detoxification, efflux transport, and estrogenic signalling). The spheroids resembled in vivo liver features and demonstrated a morphological and functional time window stabilisation from the 12th to the 20th day in culture. The model is promising for investigating fish hepatic adaptive and toxicological responses to xenobiotics.

**Abstract:**

Mammal hepatocyte spheroids have been investigated as alternative experimental models in several contexts, since three-dimensional (3D) systems have shown the potential to mimic in vivo scenarios. The description of fish hepatocyte 3D models is still minimal. This study intends to further characterize brown trout primary hepatocyte spheroids at distinct time points up to 25 days in culture. Viability, biometry, histomorphology, and basal expression of a selection of genes (metabolism and detoxification, efflux transport, and estrogenic signalling) were considered. The gene expression of whole liver samples from the same fish donor were evaluated concurrently. After 12 days in culture, the hepatocyte spheroids exhibited biometric and morphological stability. From the 12th to the 20th day in culture, the basal expression levels for most of the selected genes did not vary. The targeted mRNA levels were higher in brown trout liver samples compared to hepatocyte spheroids. Despite that, data supported that this model resembles some in vivo features. As an experimental alternative model, it showed potential to be used in a stable time window that can be exploited for exposure tests to different xenobiotics, namely, estrogenic compounds.

## 1. Introduction

The liver is a pivotal multifunctional organ, and, therefore, there has been an increasing emergence of three-dimensional (3D) hepatic models from primary cells [1,2,3,4] and cell lines [5,6,7]. Those alternative models seem to be valid approaches to test drug-induced hepatotoxicity [8,9], liver metabolism [2], and even impacts of infectious diseases, such as the severe acute respiratory syndrome coronavirus 2 (SARS-CoV-2) [10]. In contrast to two-dimensional (2D) cultures, 3D hepatocyte cultures have shown excellent cell viability and morphological and functional stability over extended culture time, demonstrating their in vivo mimicking [1,11]. Bell et al. [8] confirmed using proteome analyses that the molecular phenotypes of primary human hepatocyte (PHH) spheroids and 2D cultures were distinct. Compared to freshly thawed cells, 2.7% of the proteins (at day 14) were significantly lower in PHH spheroids, while 6% of the total proteins were lower in 2D cultures. It was also reported that hepatic transcripts in PHH spheroids were similar to those of freshly isolated cells [12]. Consequently, PHH spheroids already showed a higher sensitivity in predicting the toxicity of compounds when compared to other in vitro models, such as hepatic cell lines [12]. Further, in PHH spheroids, the intrinsic clearance of different compounds and the ability to form metabolites closely mimicked what happened in vivo [2].

Hepatocyte functionally could be maintained in PHH spheroids aged more than 7 days [11], expressing stable phase I cytochrome P450 enzymes (e.g., CYP1A2, CYP2C9, CYP2D6, and CYP3A4) until 14 and 21 days in culture [2,8]. Further, phase II metabolization enzymes (e.g., glutathione S-transferase theta 1—GSTT1 and UDP-glucuronosyltransferase family 1 member A1—UGT1A1) and phase III transporters (e.g., bile salt export pump (BSEP) transporter—ABCB11) also had high expression levels in PHH spheroids [11,12]. Other liver markers in PHH spheroids have been described, such as the steady albumin secretion [1] and the detection of the hepatocyte nuclear factor 4 alpha (HNF4α) [13]. In addition, the liver phenotype in PHH spheroids has also been assessed by parameters that reflect the re-establishment of cell compactness (e.g., increase in E-cadherin expression), polarity, and bile canaliculi formation (e.g., localization of transporters, such as multidrug resistance-associated protein 2—MRP2, MRP3, and organic cation transporter 1—OCT1) [14].

Although some of the first studies using rat and human hepatocyte spheroids, e.g., [15,16], did not appear long before those with fish [17], the mammal models have received far more attention in terms of morphological and, more importantly, functional characterization. The morphological stabilization of primary rainbow trout (*Oncorhynchus mykiss*) hepatocyte spheroids was described after 6 to 10 days in culture [3,4]. Data showed viable cells with ultrastructural characteristics of hepatocytes in rainbow trout liver cell line RTL-W1 spheroids after 15 days [18]. As reviewed by Alves et al. [19], few studies have tested the metabolic functionality of liver spheroids in fish. Despite this, fish hepatocyte spheroids have shown potential as an alternative model for future in vitro testing due to their metabolic competence and higher expression of hepatic transcripts compared to 2D cultures [4,20,21]. Specifically, mature spheroids (>10 days) from primary rainbow trout hepatocytes expressed metabolism and efflux transporter genes at levels comparable to fresh hepatocytes [4]. Further, in primary rainbow trout hepatocyte spheroids (8 days), the albumin and glucose releases—used as liver-specific markers—were higher than in monolayer cultures [3]. In the zebrafish (*Danio rerio*) liver (ZFL) cell line, vitellogenin (Vtg) and urea synthesis also increased during the 28 days in culture, which did not happen in 2D [7].

Previously, our group established a 3D in vitro model using brown trout (*Salmo trutta*) primary hepatocytes, which was maintained in culture for 30 days [22]. Morphological stabilization of hepatocyte spheroids was reached on the 12th to 16th day in culture, and 96 h exposures to 5α-dihydrotestosterone (DHT) caused distinct modulations in lipid-related targets [22]. However, this model is not well-characterized. Based on the described background, the main aims of this study were (1) to provide a basal gene expression follow-up (at the 8th, 12th, 16th, 20th, and 25th days in culture) of primary brown trout spheroids that attest to their liver-mimicking properties; (2) to compare the consistency of the basal expressions in spheroids and fresh liver samples from the same fish. Biometry, morphology, and viability of spheroids were used to complement the characterization. Some of the genes chosen are involved in liver xenobiotic metabolism and detoxification (CYP1A, CYP3A27, GST, UGT, and catalase—Cat) and transport (BSEP, multidrug resistance protein 1—MDR1, and MRP2). In addition, three estrogen exposure marker genes (VtgA, estrogen receptor α—ERα and zona pellucida glycoprotein 2.5—ZP2.5), which are expressed in juvenile brown trout under control conditions [23,24,25], were included to support the model’s application in assessing hepatotoxic effects of endocrine disruptors.

## 2. Materials and Methods

### 2.1. Fish

Brown trout juveniles (1 year old) were acclimatized upon arrival from the Aquaculture Station of Torno (Amarante, Portugal) for at least 4 weeks before experiments. Fish had a mean (±standard deviation) weight of 50.2 (±13.1) g and a total length of 17.1 (±1.8) cm. The photoperiod was set to 12 h light/12 h dark, and fish were fed daily (Trout Plus 4, AquaSoja), except before isolations. Water parameters were checked weekly and maintained with the following values [mean (±standard deviation)]: ammonium and ammonia—0.0 (±0.0) mg/L, nitrates—39.2 (±5.3) mg/L, nitrites—0.06 (±0.03) mg/L, oxygen (O_2_)—88.4 (±2.8)%, pH—8.1 (±0.2), and temperature—19.2 (±1.1) °C.

### 2.2. Hepatocyte Isolation

Ethylene glycol monophenyl ether (Merck KGaA, Darmstadt, Germany) at 0.6 mL/L was used to euthanize the fish. There were no experimental procedures, and the humane killing was made by a legally certified researcher in accordance with the Portuguese Decree-Law No. 113/2013, implementing EU Directive No. 2010/63 on animal protection for scientific purposes.

The liver of each fish was weighed, and a fragment of approximately 16 mg was sampled, snap-frozen in liquid nitrogen, and stored at −80 °C. Then, hepatocytes were isolated from the remaining liver following a two-step collagenase perfusion protocol, initially described in two classic references for isolating primary trout hepatocytes [26,27], and later adapted for brown trout [23]. Upon isolation, the mean cell viability was 80.4% (minimum: 70%, maximum: 94%) and was measured in an automatic cell counter (Countess, Invitrogen^TM^, California, CA, USA), using a 1:1 dilution of cell suspension and trypan blue 0.4% (Invitrogen^TM^).

### 2.3. 3D Cultures

Primary hepatocytes were isolated from 5 fish (allowing 5 independent in vitro experiments), and 3 technical replicates (3 plates) were used per trial. Cells were plated in Dulbecco’s modified Eagle medium/nutrient mixture F-12 (DMEM/F-12) (GE Healthcare Life Sciences, IL, USA) with 10% charcoal-stripped fetal bovine serum (FBS) (Merck KGaA, Darmstadt, Germany), 15 mM of 2-[4-(2hydroxyethyl)1-piperazinyl]-ethanesulfonic acid (HEPES) (Merck KGaA, Darmstadt, Germany) and 10 mL/L of antibiotic antimycotic solution (100×), stabilized with 10,000 units penicillin, 10 mg streptomycin, and 25 μg amphotericin B per mL (Merck KGaA, Darmstadt, Germany), in non-tissue culture-treated sterile 6-well plates (351146, Falcon, Corning, New York, NY, USA), at a cell density of 5 × 10^5^ cells/mL (total volume of 3 mL/well). This culture medium proved suitable for obtaining 3D cultures of primary hepatocytes from brown trout [22]. Incubation occurred at 18 °C, without additional supply of O_2_/CO_2_ and at constant orbital agitation (~100 rpm) (IKA^®^ MTS 2/4 digital microtiter shaker, Staufen, Germany). Under the described conditions, hepatocytes aggregate, generating spheroids over time in culture. A total of 1.5 mL of old medium was exchanged every other day with fresh medium. Sampling was performed on the 8th, 12th, 16th, 20th, and 25th days post-isolation (except for two fish, in which it was impossible to obtain enough spheroids on the last day). Spheroid manipulation during sampling was always carried out with a P100 micropipette. According to the experimental design, 3 replicates/sampling day (3 wells, each from a different plate) were considered per each independent assay.

### 2.4. Spheroids Biometric Analysis

Spheroids were photographed using an Olympus CKX41 light microscope (Tokyo, Japan) with a Pixelink M5C-CYL-PL-D685CU digital camera under a 10× objective lens. Photos (*n* = 30 spheroids/sampling day) were analysed using the AnaSP software to obtain the equivalent diameter, area, and sphericity of spheroids [28], as implemented previously [22].

### 2.5. Lactate Dehydrogenase (LDH) Assay

The LDH assay was used as an indicator of cell membrane integrity. The LDH activity was assessed in the cell culture supernatant using the LDH Cytotoxicity WST Assay kit (Enzo Life Sciences, ENZ-KIT157, New York, NY, USA) in 96-well microplates (non-tissue culture-treated plate, 351172, Falcon, Corning, New York, NY, USA). On each sampling day, 12 background controls were performed by adding 100 µL of fresh culture medium to each well. Samples corresponded to 100 µL of cell culture supernatants (always collected before medium changes) in each well, previously centrifuged at 1500 rpm (239 RCF), for 5 min. A total of 12 supernatant medium samples (*n* = 4 from each well) were measured per sampling day. Then, 100 μL of the working solution was added to all wells, and incubation lasted 30 min at room temperature, protected from light. Reactions were stopped, and the absorbance was measured at 490 nm with a Multiskan^TM^ GO microplate spectrophotometer (Thermo Scientific, Vantaa, Finland). Background subtraction was performed, and absorbances were plotted over time in culture.

### 2.6. Resazurin Assay

The resazurin assay was used to infer cell metabolic activity in spheroids over time. For each independent experiment, a minimum of 3 spheroids were collected per sampling time and transferred individually to a 96-well plate (non-tissue culture-treated plate, 351172, Falcon, Corning, New York, NY, USA). After that, 90 µL of fresh culture medium was added to each well. A stock solution of 2.2 mM of resazurin (Cayman Chemical Company, Ann Arbor, MI, USA) was prepared in sterilized PBS (1×), and a final concentration of 44 μM per well was obtained by dilution [29]. A concentration of 10 µM of resazurin was used in preliminary testing, but it proved ineffectual due to low sensitivity in the case of small spheroids. Twelve blank wells were included by adding the same amount of medium and resazurin in the wells but without spheroids. The plates were incubated at 18 °C for 3 h, at constant agitation (~100 rpm) and protected from light. We also incubated for 6 h in addition to 3 h; however, there were no significant variations in the measurements. Fluorescent quantification at 550 nm and 588 nm (excitation and emission lengths, respectively) were read with a microplate Biotek Synergy™ HTX multimode reader (Agilent, Santa Clara, CA, USA) with the software Gen5 (Agilent, Santa Clara, CA, USA). RFU values for each sample were adjusted by blank subtraction and plotted over time.

### 2.7. Spheroid Morphology

For each experiment, at each sampling day, 6 spheroids were placed individually into 1.5 mL microtubes, and fixation was carried out at room temperature, using 500 µL of 10% neutral buffered formalin (Epredia, Breda, The Netherlands). After 24 h, formalin was changed to 70% ethanol. Afterward, the spheroids were embedded in Richard-Allan Scientific HistoGel (HG-4000, Epredia, Breda, The Netherlands). A 12 h routine histological procedure was performed in an automatic processor (TP 1020, Leica Biosystems, Wetzlar, Germany), and the samples were embedded in paraffin (Histoplast, Epredia, Breda, The Netherlands) using an embedding station (EG1140C, Leica Biosystems, Wetzlar, Germany). Paraffin blocks (1 spheroid/fish per sampling time) were sectioned at 3 μm (5 sections/slide) in a fully automated rotary microtome (RM2255, Leica Biosystems, Wetzlar, Germany). Sections were stained with hematoxylin and eosin (H&E) and photographed with a light microscope (BX50, Olympus, Tokyo, Japan) with a digital camera (EP50, Olympus, Tokyo, Japan).

### 2.8. RNA Extraction and cDNA Synthesis

On each sampling day, pools of spheroids were collected as previously detailed [22], centrifuged at 1500 rpm (239 RCF) for 5 min, and the pellets were snap-frozen in liquid nitrogen and stored at −80 °C. The total RNA extraction from spheroids and liver samples was carried out using an illustra^TM^ RNAspin Mini RNA isolation Kit (GE Healthcare, Chicago, IL, USA), according to the manufacturer’s recommendations. The protocol included a DNase I treatment step to avoid genomic DNA contamination of samples. RNA purity and quantification were checked in a Multiskan^TM^ GO microplate spectrophotometer (Thermo Scientific, Vantaa, Finland), using a μDrop™ Plate, with a SkanIt Microplate Reader software (Thermo Fischer Scientific). The λ 260/280 nm ratio was 2.2 ± 0.1 (mean ± standard deviation) for all samples, which is acceptable for a pure RNA sample (~2.0) [30]. Agarose gel with GelRed (Biotium, Fremont, CA, USA) staining allowed the RNA qualitative assessment. cDNA syntheses of spheroid and liver samples were made using an iScript™ Reverse Transcription Supermix kit (Bio-Rad, Hercules, CA, USA) for a total volume of 20 μL, using 300 ng of total RNA.

### 2.9. Quantitative Real-Time Polymerase Chain Reaction (RT-qPCR)

RT-qPCR was performed in 96-well plates using a CFX Connect real-time PCR detection system with CFX Manager software (Bio-Rad, Hercules, CA, USA). SYBR Green reactions had 10 μL of iQ™ SYBR^®^ Green Supermix (Bio-Rad, Hercules, CA, USA), 5 μL of cDNA (diluted 1:5), and 200 nM of each primer and water, for a total volume of 20 μL. The cDNA inputs for spheroids and liver samples were 15 ng/reaction. Duplicates of cDNA samples and no-template controls were analysed. The product specificity was checked by performing a melt curve. The Pfaffl method was used for relative quantification [31]. Reference gene normalization was made with the geometric mean of elongation factor-1 alpha (ef1α) and β-actin (β-act), the most stable reference genes according to the NormFinder algorithm [32]. Target genes included BSEP, Cat, CYP1A, CYP3A27, ERα, GST, MDR1, MRP2, UGT, VtgA, and ZP 2.5. RT-qPCR conditions and primer sequences are shown in Table 1. Amplification efficiencies between 90% and 110% were considered acceptable.

### 2.10. Statistical Analyses

The Past 3 software, version 3.25, was used for statistical analyses [39]. All graphs were obtained with GraphPad Prism 8. A one-way analysis of variance (ANOVA) followed by the Tukey’s pairwise comparisons post hoc test was used for comparing group means. Before the ANOVA, the normality and homogeneity of data were checked by Shapiro–Wilk and Levene’s tests, respectively. When assumptions were not met, even after data transformation, the non-parametric Kruskal–Wallis ANOVA was applied, followed by the Mann–Whitney pairwise comparisons post hoc test, with sequential Bonferroni corrections. The differences were considered significant for *p* < 0.05.

## 3. Results

### 3.1. Biometry

**Figure 1:**
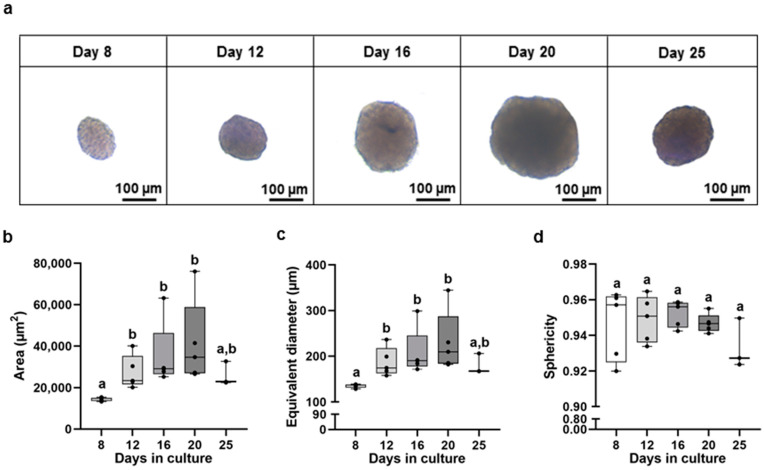
Representative bright-field images (**a**) and biometric parameters ((**b**)—area, (**c**)—equivalent diameter, and (**d**)—sphericity) of primary hepatocyte brown trout spheroids at distinct days in culture (total of 5 independent fish, *n* = 30 spheroids/day/fish, except for 2 fishes at the 25th day). Data correspond to median, minimum, maximum, and 25th and 75th percentiles. Dots indicate each fish. Days not showing common letters differ significantly (a vs. b = *p* < 0.05).

Under agitation, and when plated in non-tissue culture-treated plates, primary hepatocytes tend to self-aggregate and form spheroids that are initially smaller, heterogeneous, and less compact (until day 6, Supplementary Figure S1). During the culture period (up to 25 days), the spheroids became more compact and spherical, resulting in changes in their biometry, as shown in Figure 1a. The area of spheroids significantly increased until the 20th day in culture (the highest median area value was 34,708 μm^2^), but values generally stabilised from the 12th until the 25th day (Figure 1b).

As to the equivalent diameter, the lowest median was obtained at day 8 (137 μm), while, from the 12th until the 25th day, the median was 188 μm (Figure 1c).

For sphericity, no differences were noted in culture (Figure 1d). From the 8th to the 20th day, sphericity was always higher than 0.92. On the 25th day, the median sphericity seemed to decrease, but without significance, compared to the other days in culture.

### 3.2. Viability—LDH and Resazurin Assays

**Figure 2:**
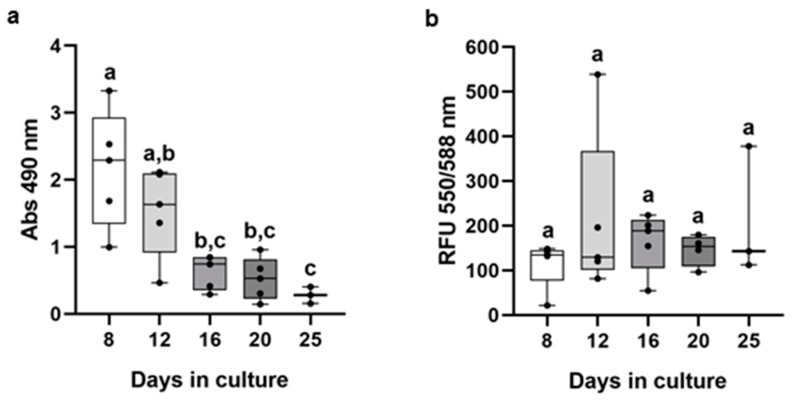
Lactate dehydrogenase (LDH) (**a**) and resazurin (**b**) data from primary hepatocyte brown trout spheroids at distinct days in culture. For LDH (total of 5 independent fish, *n* = 12 supernatant medium samples/day/fish, except for 2 fishes at 25th day) and resazurin (total of 5 independent fish, a minimum of 3 spheroids/day/fish, except for 2 fishes at the 20th and 25th day) absorbance (Abs at 490 nm) and relative fluorescence units (RFU 550/588 nm), respectively, were plotted against days in culture. Data correspond to median, minimum, maximum, and 25th and 75th percentiles. Dots indicate each fish. Days not showing common letters differ significantly (a vs. b; a vs. c; b vs. c = *p* < 0.05).

In the LDH assay, the highest LDH release was obtained on the 8th day and the lowest on the 25th day in culture. LDH leakage to the culture medium was significantly higher on the 8th and 12th days compared to the 25th day. Overall, data indicate a time-dependent decrease in LDH leakage (Figure 2a).

For the resazurin assay, the RFU values did not vary significantly over the days in culture (Figure 2b). A high variability in the RFU values was observed on the 12th and 25th days in culture.

### 3.3. Morphology

**Figure 3:**
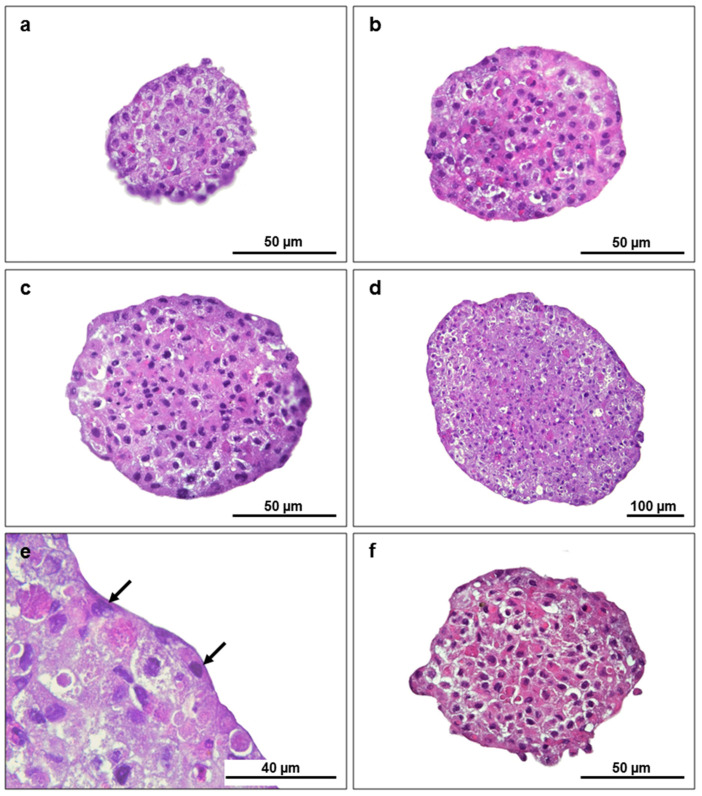
Primary hepatocyte brown trout spheroid sections stained with H&E at distinct days in culture: (**a**) 8th day; (**b**) 12th day; (**c**) 16th day; (**d**,**e**) 20th day and (**f**) 25th day. Arrows indicate epithelioid cells.

An increase in the size and compactness of spheroids was noted over time (Figure 3a–f). Cells were intact in all stages of the spheroid’s maturity, with distinct basophilic nuclei and a polyhedral hepatocyte-like shape. There was no evidence of other liver cellular phenotypes than hepatocytes. However, the spheroids were externally surrounded by a well-defined flattened layer of epithelioid cells, particularly those from the 12th to 20th days (Figure 3b–e). There was no evidence of a necrotic centre until the 25th day.

### 3.4. RT-qPCR

**Figure 4:**
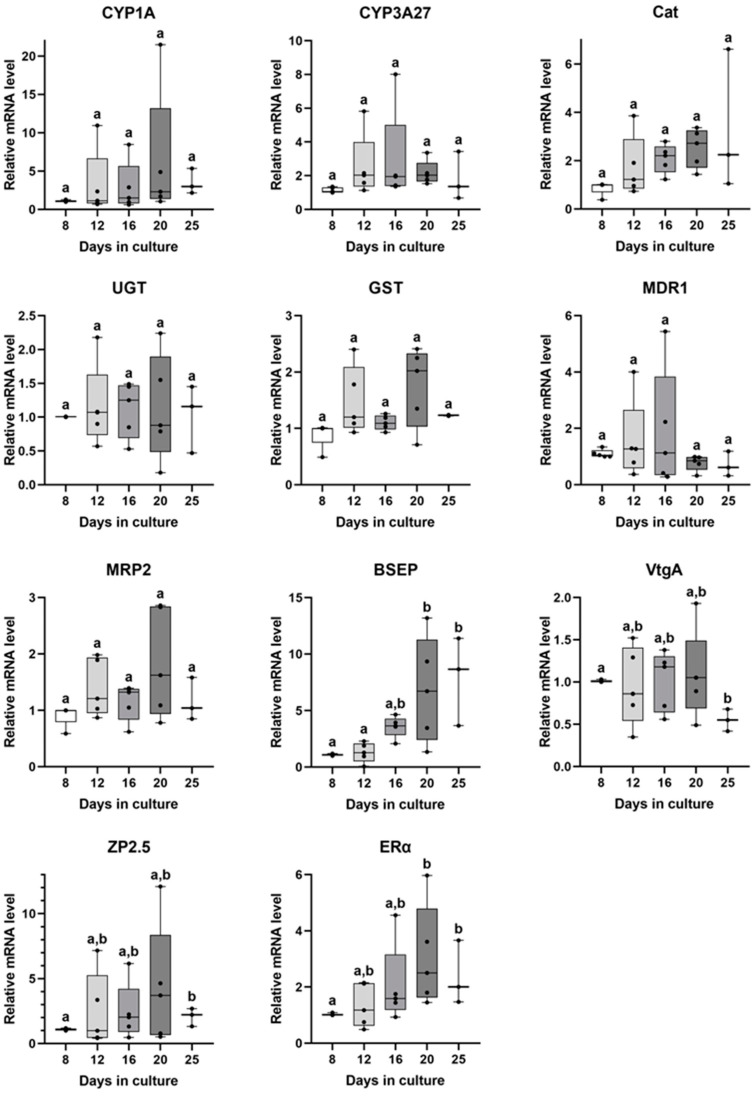
Relative mRNA levels of metabolization/detoxification, efflux transporters, and estrogenic target genes in primary hepatocyte brown trout spheroids at distinct days in culture (*n* = 5 independent fish/day, except at the 25th day). Data correspond to median, minimum, maximum, and 25th and 75th percentiles. Days not showing common letters differ significantly (a vs. b = *p* < 0.05). Dots indicate each fish. CYP1A—Cytochrome P450 1A; CYP3A27—Cytochrome P450 3A27; Cat—Catalase; GST—Glutathione S-transferase; UGT—UDP–glycosyltransferase; MDR1—Multidrug resistance protein 1; MRP2—Multidrug resistance-associated protein 2; BSEP—Bile salt export pump; VtgA—Vitellogenin A; ZP2.5—Zona pellucida glycoprotein 2.5; and ERα—Estrogen receptor alpha.

Relative mRNA levels of the selected xenobiotic metabolism, efflux transport, and estrogenic genes in primary brown trout hepatocyte spheroids are shown in Figure 4. For the xenobiotic metabolism and detoxification targets, no significant differences were displayed in the mRNA levels of CYP1A, CYP3A27, Cat, UGT, and GST. For the efflux transporters, both MDR1 and MRP2 showed stable mRNA levels along the different days in culture, while BSEP mRNA levels were significantly higher on the 20th and 25th days compared to the 8th and 12th days. Regarding the estrogenic target genes, both VtgA and ZP2.5 mRNA levels were mostly stable between the 12th and 20th day. Further, the VtgA mRNA levels were significantly down-regulated on the 25th day compared with the 8th day, while ZP2.5 mRNA levels were up-regulated on the 25th versus the 8th day. The ERα mRNA levels also showed a stability period between the 12th and 20th day, but significantly higher levels were found on the 20th and 25th days compared with the 8th day. There was a high variability for some genes at specific days in culture (e.g., VtgA, ERα, ZP2.5, CYP1A, BSEP); in 8 of 11 genes (CYP1A, UGT, GST, MRP2, BSEP, VtgA, ZP2.5 and ERα), the variability tended to be highest on the 20th day.

### 3.5. Expression Levels in Whole Liver Samples vs. Hepatocyte Spheroids

**Figure 5:**
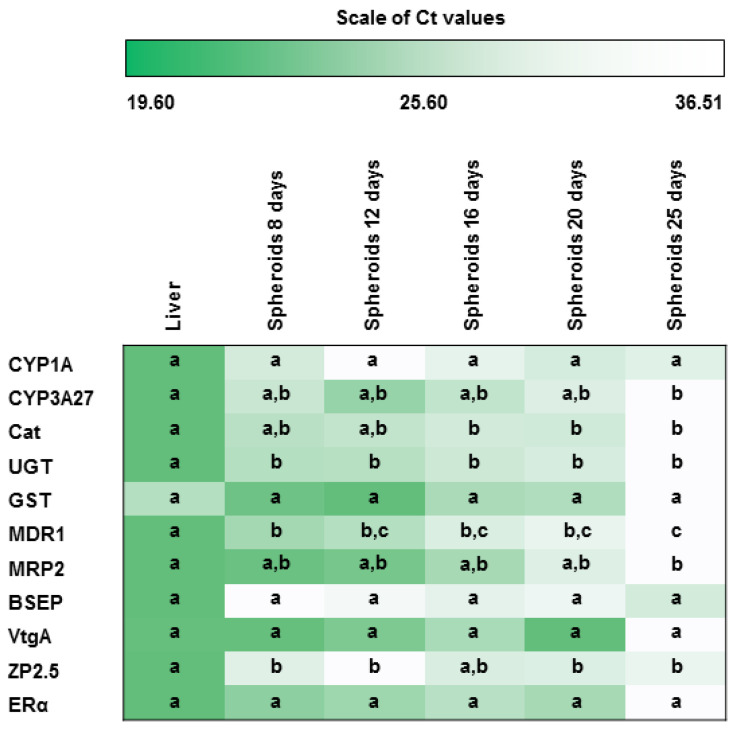
Brown trout whole liver samples vs. primary hepatocyte spheroids expression levels of metabolization/detoxification (CYP1A, CYP3A27, Cat, GST, and UGT), efflux transporters (BSEP, MDR1, and MRP2) and estrogenic (VtgA, ERα, and ZP2.5) target genes (*n* = 5 independent fish/day, except at 25th day). The maximum Ct value (lowest level of expression) is shown in white, and the minimum Ct value (highest level of expression) is shown in dark green. Dots indicate each fish. For each gene, conditions not showing common letters differ significantly (a vs. b; a vs. c; b vs. c = *p* < 0.05). CYP1A—Cytochrome P450 1A; CYP3A27—Cytochrome P450 3A27; Cat—Catalase; GST—Glutathione S-transferase; UGT—UDP–glycosyltransferase; MDR1—Multidrug resistance protein 1; MRP2—Multidrug resistance-associated protein 2; BSEP—Bile salt export pump; VtgA—Vitellogenin A; ZP2.5—Zona pellucida glycoprotein 2.5; and ERα—Estrogen receptor alpha.

In general, lower expression levels and much higher variability were noted in primary hepatocyte spheroids than in whole liver samples (Figure 5). The expression levels of CYP1A, GST, BSEP, VtgA, and ERα did not change significantly in spheroids during the days in culture or from liver samples. All target genes showed stable expression in spheroids between the 8th and 20th days. On the 25th day, spheroids had lower expressions for all genes except CYP1A, BSEP, and ZP2.5. For all the remaining genes, spheroids presented the closest pattern to in vivo expressions from the 8th to the 20th day.

## 4. Discussion

This study continues the first development and morphological characterization of spheroids from brown trout primary hepatocytes [22]. Here, the focus was on assessing the basal expression of target genes involved in xenobiotic metabolism, transport, and estrogenic signalling and its comparison with whole liver samples from the donor fish. 

Over the culture days, primary brown trout hepatocyte spheroids increased in size and compactness, achieving elliptical or spherical shapes with well-defined limits. Those characteristics were previously reported as putative markers of morphological maturity in primary rainbow trout hepatocyte spheroids [3,4]. The hepatocyte 3D structures had a defined outer layer that resembled what has been described in rat hepatocyte spheroids as “epithelial-like cells” [40]. The primary brown trout hepatocyte spheroids showed biometric and morphological stability, particularly after 12 days in culture, which supports our prior findings for the same 3D model derived from individuals from a different fish batch [22]. Previous investigations using primary rainbow trout hepatocyte spheroids have noted morphological stability a few days earlier [4]. Despite the stabilization period (12th to 20th day), the area and equivalent diameter showed a high variability in values each day because not all spheroids formed with the same kinetics. Above all, the cell aggregation process differs between spheroids and can be influenced by the fish donor. On the 12th day in culture, primary hepatocyte spheroids had a median equivalent diameter of 175 µm, which did not vary until the 25th day. Although Langan et al. [41] suggested that spheroids should have a diameter of less than 150 µm, it is known from mammalian models that certain factors, such as spheroid compactness and shape, as well as the method used to form spheroids [42,43,44], can affect how nutrients and oxygen diffuse into the inner cell core, making the diameter a restrictive parameter to consider per se. Irrespective of their size, the histological analysis of the spheroids generated in this study did not demonstrate cellular alterations compatible with the formation of a necrotic core, as we have seen for the same model when maintained under distinct culture conditions [22]. Resazurin and LDH release were used to estimate cell viability, providing a proxy of the mitochondrial functionality of the hepatocytes within the spheroids and their membrane integrity, respectively. The resazurin assay did not show changes in RFU values, which suggests that hepatocytes remain metabolically active in 3D cultures. Nevertheless, this assay had a limited sensitivity since smaller spheroids (having fewer cells) gave some inconsistent readings. Further adjustments to the protocol (concentration and/or incubation time) or an alternative and more robust assay should be considered in future studies. The LDH release significantly decreased from day 8 to days 16–25, indicating that mature spheroids (at least from the 16th day) could maintain membrane integrity and prevent LDH leakage. In agreement, it has been shown that mature spheroids retain higher levels of intracellular LDH than immature spheroids, making LDH leaking a reliable marker of cell membrane integrity [3]. Overall, comparing the earlier and later days in culture, data from the LDH, as well as morphological and biometric parameters of primary hepatocyte spheroids, suggest that spheroids reach structural maturity at 12–16 days, potentially better mimicking the in vivo model.

Earlier studies with mammal hepatocytes demonstrated that the mRNA levels of liver-specific genes drastically decreased in primary monolayer cultures but not in rat hepatocyte spheroids [45] or mouse liver slices [46]. Thus, analysing liver-specific gene regulation in primary hepatocyte brown trout spheroids at different stages of culture can provide new insights into hepatocyte functionality because transcriptions are significantly influenced by tissue organization. Overall, the basal expression levels of the selected genes in brown trout primary hepatocyte spheroids showed stable levels between the 12th and the 20th days in culture, corresponding to the morphological stability period found in this study. Our findings are consistent with Flouriot et al. [17], which evidenced variable mRNA levels during the early stage of rainbow trout hepatocyte spheroids aggregation (up until the 8th day). In the same way, Uchea et al. [4] also noted higher stability of gene expression in mature hepatocyte spheroids (over 10 days in culture). In the same vein, Messner et al. [47] noted that the major transcriptome and proteome changes in human microtissues (obtained from primary human hepatocytes co-cultured with non-parenchymal cells) also occurred during an initial repair/recovery stage (~7 days).

Here, brown trout liver gene expression was generally higher than in hepatocyte spheroids (from the same individuals). Different factors can explain that fact. First, brown trout hepatocyte spheroids do not mimic the entire liver-like environment, since non-hepatocytic cells—identified as significant regulators in co-cultures with hepatocytes [48,49]—were not included in our model. Second, the liver dissociation process causes rapid changes in mRNAs [50], which may explain, at least partially, the different expression levels in spheroids versus the liver. Finally, Fischer et al. [51] reported that the absence of specific substrates in cultures might also lead to a decrease in mRNAs in rainbow trout permanent cell lines, for example, for some liver efflux transporters. Despite that, the outcomes from this study showed that the metabolism, detoxification, and transport pathways are still active in the brown trout hepatocyte spheroids, during a longer time window than commonly found in 2D cultures.

Regarding the expression of metabolization/detoxification genes (CYP1A, CYP3A27, Cat, GST, and UGT), stable levels were obtained during the culture time, although a high variability was found for some genes and days in culture. CYP1A2 and UGT1A1 expressions were also constant in PHH spheroid cultures [11,14] for over 20 days. In rainbow trout hepatocyte spheroids, the CYP1A expression levels were the highest at initial times in culture (5 and 7 days), while UGT expression levels increased in mature spheroids [4]. On the 15th day in culture, rainbow trout liver cell line (RTL-W1) spheroids expressed CYP1A mRNA levels higher than monolayer cultures and were highly inducible with β-naphthoflavone [18]. These data indicate the possibility of using our 3D model in metabolization experiments, although this will need to be verified using different inducers.

Trout liver expression of the selected ATP binding cassette (ABC) efflux transporters (BSEP–ABCB11, MRP2–ABCC2, and MDR1–ABCB1) were previously described in other studies [52,53]. In mammals, BSEP is the main bile salt secretor, while MRP2 and MDR1 are responsible for transporting many xenobiotics, endogenous compounds, and their metabolites [54,55]. Immunohistochemistry has previously demonstrated hepatocyte polarisation in human spheroids (BSEP and MRP2) [47]. Interestingly, in our study, the highest BSEP mRNA levels were found in mature brown trout hepatocyte spheroids, indicating that the intrahepatic biliary canalicular network may have been restored (at least partially) in these 3D structures. Overall, our data corroborate the BSEP expression profiles found in hepatocyte rainbow trout spheroids, in which there was an initial stabilization of expression levels (7–15 days), with a subsequent increase after 25 days in culture [4]. Across all transporters tested in this study, BSEP consistently showed greater mRNA levels in spheroids cultured for longer than 16 days. Zaja et al. [52] also reported that BSEP expression was higher than MDR1 and MRP2 in rainbow trout liver and primary hepatocytes [52]. Further, higher BSEP expression than other efflux transporters was also found in rainbow trout hepatocyte spheroids [4]. On the contrary, low mRNA and protein activities of BSEP were described in hepatocyte-derived cell lines (RTL-W1 and R1) [51]. Overall, the evidence points out that, compared with continuous cell lines, the primary cultured hepatocyte spheroids more accurately reflect the phenotypic and mRNA expression of BSEP in vivo. Finally, the MRP2 and MDR1 mRNA levels in brown trout hepatocyte spheroids remained stable during the 25 days in culture, which does not exclude that they may be induced under stimulation of specific substrates, but this conjecture deserves future investigation. Contrarily, in rainbow trout hepatocyte spheroids, the expression levels of MRP2 and MDR1 significantly increased on the 25th day of culture [4], suggesting that distinct inter-species physiological regulations can exist.

Estrogenic target genes displayed stable mRNA levels between the 12th and 20th day, demonstrating the potential of brown trout primary hepatocyte spheroids, during that period, for testing the effects caused by different disruptors of the estrogenic pathway. In spheroids obtained from zebrafish liver (ZFL) cell line, Vtg (i.e., vtg1 and vtg5), and ER (i.e., esr2a) mRNAs were more highly expressed than in 2D cultures, which reiterates the advantage of 3D models for testing these pathways [7]. In accordance, Flouriot et al. [17] reported constant basal expression of ER mRNA levels in rainbow trout hepatocyte spheroids during 30 days in culture, and those levels were induced after estradiol stimulation [17]. In rainbow trout hepatocyte spheroids, the Vtg expression under control conditions was insignificant for 21 days [17], but, contrary to this, in ZFL spheroids, the levels were detectable and the Vtg synthesis increased until day 28 [7]. In the present study, VtgA expression was maintained at detectable levels until 25 days in culture, with a decrease at day 25 (compared with day 8), meaning that it could be the turning point of VtgA expression in this 3D model without estrogenic stimulation.

Considering the five independent assays carried out, high variabilities in the mRNA levels of some genes (VtgA, ERα, ZP2.5, CYP1A, BSEP) were observed at each time in culture, suggesting an inherent “interference” (i.e., natural variability) of the donor fish. The process of spheroid formation and the culture longevity also seem to be affected by the donor fish, since we could not keep enough spheroids from two fish until the last days of culture. The literature using PHH spheroids supports our observations because metabolic drug profiles are largely distinct within spheroids obtained from different individuals [2,11]. Spheroids retain individual phenotypes, and, accordingly, Schofield et al. [56] recommended a pre-selection of donors based on spheroid formation and metabolic capacity. To our knowledge, no other studies using fish primary hepatocyte spheroids addressed this issue, but it should be considered in the design of future studies.

## 5. Conclusions

Brown trout primary hepatocyte spheroids from juvenile fish showed biochemical, morphological, and basal gene expression (i.e., metabolization, detoxification, transport, and estrogenic targets) stabilization mostly from the 12th to the 20th day in culture. The viability and functionality of this model over this time window can be further explored as a screening methodology to assess the hepatic adaptive and toxicological responses to xenobiotics in fish, from those impacting biotransformation to those impacting estrogenic compounds.

## Figures and Tables

**Table 1:** Primer sequences, annealing temperature (AT), and efficiencies (E).

Gene	Abbreviation	Primers Sequences	AT (°C)	E (%)	References
Bile salt export pump	BSEP	F: 5′-CCGACCAGGGCAAAGTGATT-3′ R: 5′-CAGAATGGGCTCCTGGGATAC-3′	60.0	93.5	[4]
Catalase	Cat	F: 5′-CACTGATGAGGGCAACTGGG-3′ R: 5′-CTTGAAGTGGAACTTGCAG-3′	58.0	91.4	[33]
Cytochrome P450 1A	CYP1A	F: 5′-GATGTCAGTGGCAGCTTTGA-3′ R: 5′-TCCTGGTCATCATGGCTGTA-3′	60.0	99.9	[4]
Cytochrome P450 3A27	CYP3A27	F: 5′-GACGGTGGAGATCAACG-3′ R: 5′-GAGGATCTCGACCATGG-3′	60.0	96.2	[4]
Estrogen receptor alpha	ERα	F: 5′-GACATGCTCCTGGCCACTGT-3′ R: 5′-TGGCTTTGAGGCACACAAAC-3′	61.6	91.2	[34]
Glutathione S-transferase	GST	F: 5′-AGCTGCTCCCAGCTGATCC-3′ R: 5′-CAAACCACGGCCACATCATGTAATC-3′	60.0	92.5	[35]
Multidrug resistance protein 1	MDR1	F: 5′-ACGTGCGCTCCCTGAACGTG-3′ R: 5′-GCGTTGGCCTCCCTAGCAGC-3′	60.0	103.6	[35]
Multidrug resistance-associated protein 2	MRP2	F: 5′-CCATTCTGTTCGCTGTCTCA-3′ R: 5′-CTCGTAGCAGGGTCTGGAAG-3′	60.0	98.5	[4]
UDP–glycosyltransferase	UGT	F: 5′-ATAAGGACCGTCCCATCGAG-3′ R: 5′-ATCCAGTTGAGGTCGTGAGC-3′	60.0	100.8	[4]
Vitellogenin A	VtgA	F: 5′-AACGGTGCTGAATGTCCATAG-3′ R: 5′-ATTGAGATCCTTGCTCTTGGTC-3′	62.9	99.0	[34]
Zona pellucida glycoprotein 2.5	ZP 2.5	F: 5′-ATCAATAACCACAGCCACAATG-3′ R: 5′-ACCAGGGACAGCCAATATG-3′	55.0	99.0	[36]
Glyceraldehyde-3-phosphate dehydrogenase	Gapdh	F: 5′-CCACCTATGTAGTTGAGTC-3′ R: 5′-ACCTTGAGGGAGTTATCG-3′	55.0	92.8	[37]
Ribosomal protein l8	rpl8	F: 5′-TCAGCTGAGCTTTCTTGCCAC-3′ R: 5′-AGGACTGAGCTGTTCATTGCG-3′	59.0	93.8	[34]
β-actin	β-act	F: 5′-TCTGGCATCACACCTTCTAC-3′ R: 5′-TTCTCCCTGTTGGCTTTGG-3′	55.0	96.1	[38]
Elongation factor-1 alpha	Ef1α	F: 5′-TGCCACACTGCTCACATC-3′ R: 5′-TCTCCAGACTTCAGGAACTTG-3′	55.0	109.0	[38]

## Data Availability

Available from the corresponding author upon reasonable request.

## Supplementary Materials

The following supporting information can be downloaded at: https://www.mdpi.com/article/10.3390/ani13142277/s1, Figure S1: Bright-field photos of brown trout primary hepatocytes at the start of culture, demonstrating the spheroid formation process.
