# Supplementary material for: A Step Forward in the Characterization of Primary Brown Trout Hepatocytic Spheroids as Experimental Models
Source: Animals (Basel). 2023 Jul 12;13(14):2277. doi: 10.3390/ani13142277 (PMC10376616; doi:10.3390/ani13142277)
Supplement: Supplementary file 1 [file animals-13-02277-s001.zip › animals-2438499-supplementary.pdf]

animals

Supplementary Materials

A step forward in the characterization of primary brown trout hepatocytic spheroids as experimental models

Rodrigo F. Alves[1,2], Célia Lopes[1,2], Eduardo Rocha[1,2] and Tânia V. Madureira[1,2,*]

[1] Team of Animal Morphology and Toxicology, Interdisciplinary Centre of Marine and Environmental Research (CIIMAR/CIMAR), University of Porto (U.Porto), Terminal de Cruzeiros do Porto de Leixões, Av. General Norton de Matos s/n, 4450-208 Matosinhos, Portugal

[2] Laboratory of Histology and Embryology, Department of Microscopy, ICBAS — School of Medicine and Biomedical Sciences, University of Porto (U.Porto), Rua Jorge Viterbo Ferreira 228, 4050-313 Porto, Portugal

* Correspondence: author: Tânia V. Madureira; Interdisciplinary Centre of Marine and Environmental Research (CIIMAR/CIMAR), University of Porto (U.Porto), Terminal de Cruzeiros do Porto de Leixões, Av. General Norton de Matos s/n, P 4450-208 Matosinhos, Portugal; E-mail: tvmadureira@icbas.up.pt

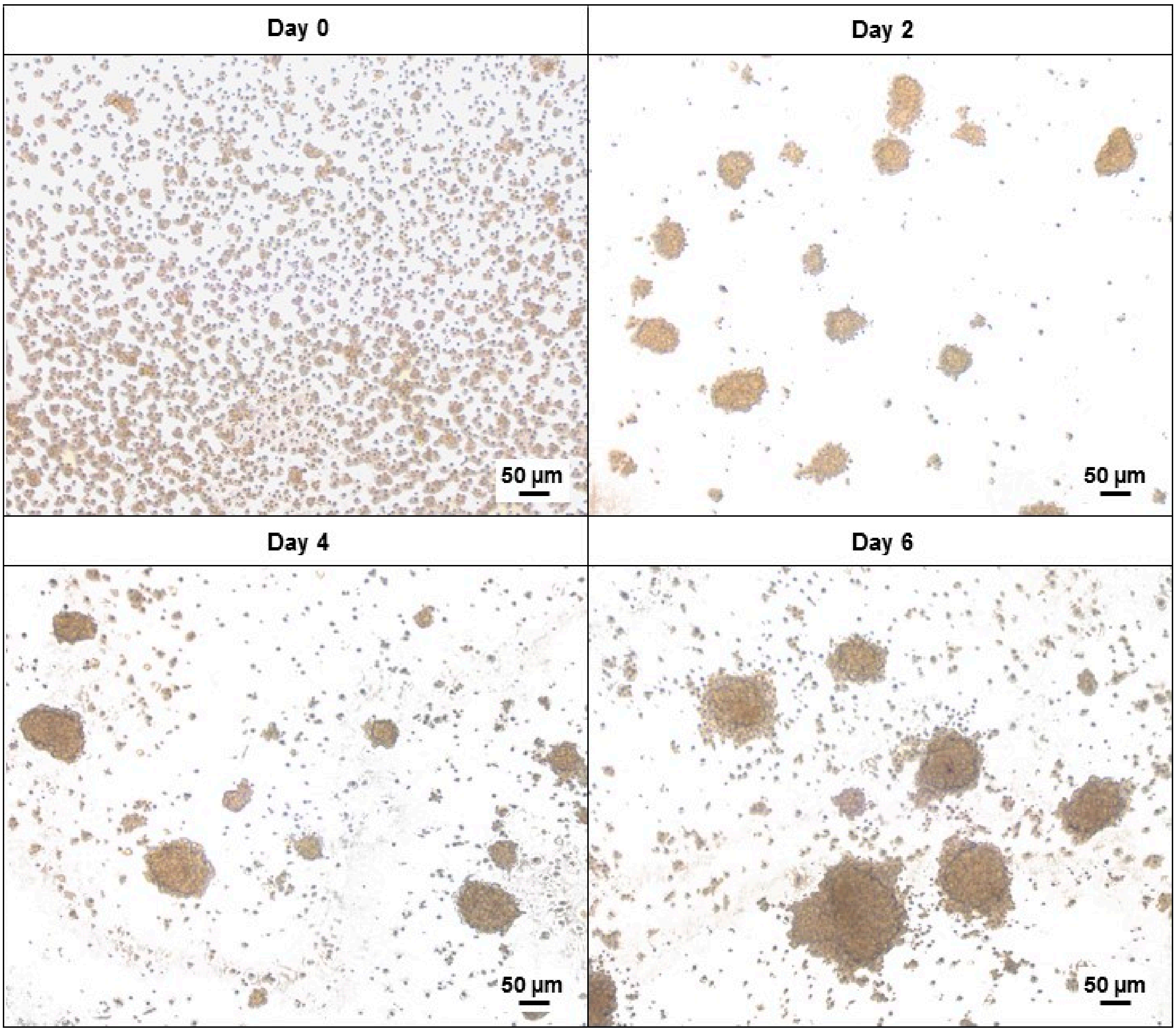

Figure S1. Bright-field photos of brown trout primary hepatocytes at the start of culture, demonstrating the spheroid formation process.
